# Recent Biomimetic Approaches for Articular Cartilage Tissue Engineering and Their Clinical Applications: Narrative Review of the Literature

**DOI:** 10.1155/2022/8670174

**Published:** 2022-04-22

**Authors:** Hamza Abu Owida

**Affiliations:** Medical Engineering Department, Faculty of Engineering, Al-Ahliyya Amman University, Amman 19328, Jordan

## Abstract

Since articular cartilage is lacking blood vessels and nerves, its capacity to heal is extremely limited. This means that ruptured cartilage affects the joint as a whole. A health issue known as osteoarthritis can develop as a result of injury and deterioration. Osteoarthritis development can be speeded up by the widespread deterioration of articular cartilage, which ranks third on the list of musculoskeletal disorders requiring rehabilitation, behind only low back pain and broken bones. The current treatments for cartilage repair are ineffective and rarely restore full function or tissue normalcy. A promising new technology in tissue engineering may help create functional cartilage tissue substitutes. Ensuring that the cell source is loaded with bioactive molecules that promote cellular differentiation and/or maturation is the general approach. This review summarizes recent advances in cartilage tissue engineering, and recent clinical trials have been conducted to provide a comprehensive overview of the most recent research developments and clinical applications in the framework of degenerated articular cartilage and osteoarthritis.

## 1. Introduction

Cartilage degeneration is turning out to be progressively problematic for physicians because of restricted self-recuperation abilities coming about because of the negligible development limit of chondrocytes, the sole cell components of cartilage, and an absence of veins, nerves, and lymphatics. The articular cartilage that covers synovial joints is the most commonly recorded cartilage deterioration. The fact that articular cartilage damage affects so many people is a major clinical concern. Hence, over time, it can add to the movement of osteoarthritis, which ranks third on the list of musculoskeletal disorders that necessitate rehabilitation, behind only low back pain and broken bones [1–3].

Besides, the fairly high commonness of articular cartilage debasement and the proclivity for osteoarthritis are exacerbated by the world's maturing populace. In 2019, there were 320.7 million cases of osteoarthritis in adults aged 30 years and above globally, and by 2028, this number is expected to rise to 367.7 million [4].

The three kinds of restorative procedures for treating articular cartilage corruption are as follows: medications to treat symptoms, restoration, and regeneration. Anti-inflammatory medications such as ibuprofen, acetaminophen, and ibuprofen are examples of indicative medicines. Restorative therapy incorporates microfracture, scraped spot, penetrating, osteochondral allograft, and mosaicplasty, and regeneration treatment incorporates autologous chondrocyte implantation and framework-initiated autologous chondrocyte implantation. Treatments for chondral and subchondral defects using restorative and regeneration techniques have been reported to be beneficial methods to restore natural articular cartilage, but they cannot completely stop the degeneration of articular cartilage, so tissue engineering approaches have been developed to address this issue. Articular cartilage tissue engineering has advanced significantly since its inception and has just been considered a potential opportunity for recovering natural articular cartilage [5–7]. A comprehensive overview of the most recent research developments and clinical applications in the framework of degenerated articular cartilage and osteoarthritis is provided in this review, which includes recent advances in cartilage tissue engineering and recent clinical trials.

**Figure 1:**
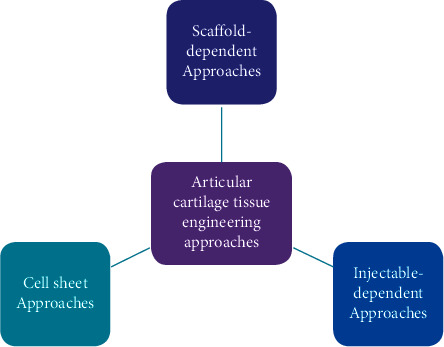
Articular cartilage tissue engineering approaches.

Through the use of articular cartilage engineering, it has been intended to produce tissue that is structurally, biochemically, and mechanically similar to natural articular cartilage tissue through the use of articular cartilage engineering. This field of research has also generated a wide range of biomaterials, biofabrication, and assessment methods [7, 8]. The following sections cover the most recent advancements in engineered cartilage tissue, including evaluations in preclinical, *in vivo*, and *in vitro* experiments, along with current clinical trials.  Figure 1 shows articular cartilage tissue engineering approaches.

## 2. Scaffold-Dependent Approaches

Scaffolds are designed to promote cellular differentiation and proliferation while also supplying chondrogenic bioactive substances, and several scaffolds have been constructed for varied goals in articular cartilage tissue creation [9, 10]. Nürnberger et al. set out to create a scaffold with a definite structure that regulates extracellular matrix production and structure within the recovery of cartilage defects. To accomplish this, they used a CO_2_ laser to imbed native articular cartilage extracellular matrix in trilayered zones. An *in vivo* model of decellularized GAG-depleted fiber shows better capacity to conduct the newly produced fibers in parallel direction, preventing unwanted irregular accumulation and thus enhancing cartilage-like tissue recovery [11].

An osteochondral defect in a rabbit model was studied using recombinant transglutaminase 4 in combination with mesenchymal stem cells derived from the synovial membrane and encapsulated in collagen, hyaluronan, and fibrinogen blend hybrids. By boosting integrin 1 expression and reorganizing actin, recombinant human transglutaminase 4 improves articular cartilage regeneration [12].

Collagen sourced from the skin of a human being could be used as a framework for articular cartilage tissue creation. A dermal collagen sheet and adipose-derived mesenchymal stem cells were integrated with collagen substrates in an experimental setup developed by Dang et al. They found that dermal collagen had a big effect on the structure and development of chondrocytes in the lab [13].

Additionally, Dufour and colleagues involved fibrin and chondrocytes that had been allowed to be treated with chondrogenic mixtures in their study. *In vivo* osteochondral damage: this model was tested first-hand both inside a laboratory setting and outside, and the results showed that self-assembling peptide scaffolds with chondrocytes were similarly efficient in patching up osteochondral damage [14].

Coculturing is another new advancement in cartilage tissue engineering techniques because cell signaling can help counterbalance articular cartilage's poor regenerative capabilities by retaining chondrocyte phenotype and boosting cartilage extracellular matrix regeneration. Owida et al. investigated an *in vitro* coculture system of mesenchymal stem cells and chondrons, using three different cultures of chondrons or chondrocytes with mesenchymal stromal cells. The coculture technique was found to be higher to single-cell cultures in terms of cartilage extracellular matrix synthesis [15]. A thermosensitive chitosan-glycerophosphate hydrogel was utilized by Scalzone et al. to construct an *in vitro* 3D scaffold for the encapsulation of bone marrow-derived mesenchymal stromal cells, and then human articular cartilage chondrocyte spheroids were added to the composite. It was found that the coculture system demonstrated promising regeneration of the cartilage, implying that mesenchymal stromal cells may play an important role in improving chondrocyte metabolic activity, which is normally lower in wounded areas. Chondrons are used in conjunction with other cells in the second type of the coculture model [16]. For their study, Duan et al. used both rabbit chondrocytes and alginate-encapsulated alginate spheres. Three different tissue engineered constructions were tested *in vivo* against chondrocytes and chondrons alone to see if the coculture method was effective in treating osteochondral lesions in the knees of white rabbit models. While collagen type II, aggrecan, and GAG were made well by the coculture method, the results were the same as when chondrocytes were grown alone [17].

Natural-based scaffolds, mainly collagen type I, are commonly used for cell-free articular cartilage repair. Szychlinska et al. evaluated collagen type I natural scaffolds in vivo using outbred rat models with femoropatellar groove cartilage lesions, and this scaffold displayed biocompatibility and efficient recruitment of host cells for articular cartilage regeneration. Collagen type I-based scaffolds have been studied before although under different experimental settings [18].

In 2020, Wang and colleagues employed biological chondrocytes-based sheet technology to produce a natural-like extracellular matrix scaffold for osteochondral repair obtained from allogeneic bone marrow mesenchymal stromal cells. To do this, cell sheets were created, decellularization was performed using sodium dodecyl sulfate, and decellularized extracellular matrix scaffolds were obtained. An osteochondral defect model in a rabbit was found to be best served by treatment with 0.5% sodium dodecyl sulfate, which was found to re-establish both healthy periosteal bone tissue and avascular articular cartilage at the same time [19]. In 2018, Dai and colleagues created a new poly(lactide-coglycolide) scaffold with microtubular pores oriented radially. Using rabbit models with osteochondral defects, *in vitro* and *in vivo* studies demonstrated that this scaffold allowed bone marrow mesenchymal stromal cells to migrate and distribute more effectively than random poly(lactide-coglycolide) scaffolds [20].

Milner et al. created a new scaffold that mimics the articular cartilage's mechanical characteristics. It was possible to fabricate and assess an *in vitro* trinetwork hydrogel comprising two biphasic double network hydrogels and a polymer, and the research showed that this hydrogel had outstanding resistance properties and could inhibit conflicting chondral injury in partial joint restoration [21]. Other researchers improved cell-free scaffolds by using them in conjunction with the delivery of bioactive chemicals. Lolli et al. loaded a microRNA inhibitor targeting miR-221 into a fibrin/HA scaffold with or without a lipofectamine carrier. Osteochondral defects heal more effectively when miR-221 infiltrating cells are suppressed using this method, especially with the lipofectamine carrier in calves with osteochondral defects *in vitro* and *in vivo* [22].

For example, Jiang et al. investigated the use of human Wharton's jelly mesenchymal stem cell-derived exosomes together with a scaffold made from extracellular matrix and porcine acellular cartilage for the recovery of articular cartilage lesions. Following the positive cytocompatibility experiments *in vitro*, the following *in vivo* models were used: human Wharton's jelly mesenchymal stem cell-derived exosomes were injected into Sprague Dawley rat models to investigate the regulation consequences upon the articular cartilage tissue, while human Wharton's jelly mesenchymal stem cell-derived exosomes were embedded in an acellular cartilage extracellular matrix scaffold implant in rabbit models to investigate the reparative effects. These tests demonstrated that human Wharton's jelly mesenchymal stem cell-derived exosomes had anti-inflammatory and osteochondral regeneration properties [23].

In 2022, Davachi et al. fabricated a hyaluronic acid/chitosan cartilage-like scaffold via horseradish peroxidase enzymatic crosslinking. *In vitro* experiments showed that mesenchymal stem cells have more likely chondrogenic potential than in control samples, implying that they have promising expression potential for cartilage-like biomarkers [24]. Huang et al. constructed a 3D-microenvironment composite comprising GelMA hydrogel with a peptide sequence PFSSTKT-modified chondrocyte extracellular matrix microspheres. The 3D-microenvironment composite was shown to be able to control the migration of rabbit bone marrow mesenchymal stem cells *in vitro*. The 3D-microenvironment composite promoted the employment of rabbit bone marrow mesenchymal stem cells from the damaged tissue two weeks after graft *in vivo*. A rabbit study using a 3D-microenvironment composite found that it was successful in regenerating healthy hyaline cartilage, as opposed to the control treatment, which mainly restored fibrous tissue instead [25].

## 3. Injectable-Dependent Approaches

Researchers are encouraged to use tools that will allow them to perform treatments that are as noninvasive as possible for articular cartilage recovery that will eventually replace invasive surgeries. It is common practice to deliver cells only to the site of the defect with the most basic injectables [26]. In 2021, Wasai and colleagues injected an allogeneic polydactyly-derived chondrocyte plug with minimally invasive surgery. The results indicate that injection of polydactyly-derived chondrocyte plug has no significant effect on the cell availability. Intra-articular injection of polydactyly-derived chondrocyte plug as a point-of-care treatment for osteoarthritis may be a feasible and less invasive method of administering the drug [27]. Takagi et al. used a rabbit model to evaluate whether weekly intra-articular injections of autologous adipose-derived mesenchymal stem cells sheets can prevent the development of osteoarthritis *in vivo*. In contrast to the control group, the supplied adipose-derived mesenchymal stem cells demonstrated protective qualities toward chondrocytes, reducing cartilage degradation and resulting in a slower development of osteoarthritis [28]. In their study, Köhnke et al. used an *in vivo* experiment (rabbit model) to assess the efficacy of adipose-derived mesenchymal stem cells injection for the treatment of temporomandibular joint osteoarthritis *in vivo*. After a month of follow-up, stem cells, particularly those implanted in hyaluronan, showed the best results in terms of articular cartilage regeneration; nevertheless, there were no statistically significant differences between the four groups when it came to tissue porosity and mineralization heterogeneity [29].

Qu et al. tested a similar formerly stated injectable, with the goal of delivering bone marrow-derived mesenchymal stromal cells via alkaline treatment using open-porous poly(lactide-coglycolide) microparticles; also, in a rat model with cartilage injury, it exhibited better cartilage defect recovery [30].

Co et al. used injectable biomolecules in protective treatment with the purpose of using click chemistry to combat posttraumatic osteoarthritis. They devised a dual-acting method for this aim, which uses polyethylene glycol to first target apoptotic chondrocytes and then deliver chondrocytes that are actively metabolizing. The results employing autogenic cartilage implanted in a pos-traumatic osteoarthritis defect indicated favorable results in cartilage damage specifically [31]. Furthermore, more advanced methods were used to improve articular cartilage regeneration using bioactive molecules. Xu et al. published a study that looked at a novel tissue engineering technique for treating osteoarthritis which modified exosomes . Osteoarthritis can be controlled through the production of mesenchymal stromal cell-binding peptides. Experiments on S rats with knee osteoarthritis show that the keratogenic transfer method is a good way to get new cartilage back in the knee [32].

Yuan et al. investigated the use of cell-free injectables in mice and rabbit models with cartilage defects. This was accomplished using a unique one-step ultrasonication crosslinking approach for the restoration of cartilage defected tissue that was both safe and effective [33]. Schaeffer et al. used Sprague Dawley rat models to test a simple acellular injectable treatment of articular cartilage injury by injecting microporous annealed particle gel into the knee joint and then photoannealing it. Microporous annealed particle hydrogels showed stable integration into defects as compared to saline injections [34]. Other researchers concentrated on using nanotechnology to create and characterize nanocomposite-injectable hydrogels.

In 2021, Tang and his team made thermosensitive poly(l, d-lactide)-polyethylene glycol-poly(d, l-lactide) hydrogels. These hydrogels were used to make composite hydrogels that could deliver platelet lysate. In *vitro* tests revealed suitable mechanical characteristics, whereas in rat experiments of osteoarthritis and osteochondral osteoarthritis revealed adequate cartilage tissue preservation, early degeneration of cartilage, and increased cartilage repair in the later stages of osteoarthritis [35]. In 2020, Wu and colleagues intended to combine a hyaluronan hydrogel as an injected-like capsule with poly(lactide-coglycolide) particles. Following the evaluation of this system in rabbit templates with full-thickness cartilage damage, the researchers concluded that the system was effective [36].

In 2022, Zhou et al. fabricated catechol-modified chitosan 3D-microenvironment hydrogel for cartilage regeneration. In a rat model, the injected hydrogel within bone mesenchymal stem cells demonstrated a promising ability promoting proliferation and chondrogenic differentiation. According to the findings of gross assessment and histology, hydrogel loaded with bone mesenchymal stem cells repaired cartilage defects better *in vivo* than the untreated group and hydrogel alone [37]. Another study by Bhattacharjee and colleagues (2022) intended to construct a minimally invasive injectable system that uses amnion membrane from the human placenta as a carrier for adipose-derived stem cells for articular cartilage injury repair. The potential for injectable hydrogels to promote cartilage tissue regeneration was demonstrated in this study; the regenerative effect of the hydrogel was comparable with the synergistic anti-inflammatory and chondrogenic effects of the injectable hydrogels to regenerate cartilage tissue in a rat osteoarthritis model [38].

## 4. Cell Sheet Approaches

The goal of cell sheet technology is to create an implantable sheet of cells that is densely packed with high-density cells coupled by a dense extracellular matrix collected without the use of biomolecules, catalyst, which is the most widely utilized technology for this purpose [39, 40].

Wongin et al. conducted one of the most recent studies on cell sheet technology, attempting to assess whether the formation of chondrocyte sheet-cancellous bone tissues is accomplished using a trichondrocyte sheet cultured on the top of cancellous bone. In rabbit models with cartilage injury, experiments were conducted for this purpose, with the latter revealing that chondrocyte sheets aid in the formation of hyaline-like cartilage and that chondrocyte sheet-cancellous bone tissues aid in osteochondral repair [41]. Takizawa et al., who transplanted cell sheets of human chondrocytes into human synoviocytes, examined chondrocytes cell sheet. These *in vivo* investigations revealed that, after 12 weeks, all groups witnessed a decrease in the number of cells, and therefore, only chondrocyte-based sheets were capable of filling the lesions with a conjunction of articular cartilage and fibrous tissue [42]. Cell sheet technology, on the other hand, has been used widely with mesenchymal stromal cells, not just differentiated cells, by using the cell sheet method. In 2020, Thorp and colleagues created cartilaginous cell sheet structures from bone marrow-derived mesenchymal stromal cells. Human articular cartilage tissue is used for verifying the chondrogenesis development, preservation of cartilaginous potential, and natural adherence to articular membrane [43]. In 2020, You and colleagues used human amniotic mesenchymal stem cells in another effort in cell sheet technology. In this study, human amniotic mesenchymal stem cells were employed to make cell sheets, which were subsequently enriched with cartilage particles before being tested in rabbit replicas with hyaline cartilage lesions. The results showed that human amniotic mesenchymal stem cell sheets—cartilage particle complexes—have promising morphological, histological, cartilage, and subchondral bone regeneration capabilities [44].  Table 1 summarizes the benefits and drawbacks of articular cartilage tissue engineering approaches.

## 5. Clinical Studies

The most recent and descriptive research on articular cartilage tissue engineering has been described previously. In spite of the fact that these studies have contributed to significant advancements in cartilage tissue recovery, turning their findings into clinical practice has been hampered by the restricted number of available clinical trials. The results of clinical investigations on cartilage tissue engineering techniques are presented as follows [45].

Zhou et al. published a recent clinical trial that has shown that the infrapatellar fat pad, a common source of mesenchymal stem cells throughout knee arthroscopy, is safe and effective. Both knee arthroscopic therapy and knee arthroplasty therapy with patient-derived infrapatellar fat pad cell concentrates were offered to patients suffering from symptoms of knee osteochondral lesions. The results confirmed that infrapatellar fat pad cell concentrates had a significant effect on lowering hurt and enhancing cartilage recovery in these individuals [46].

Hyaluronan-packed mesenchymal progenitor cells from a person's own fat were used in another clinical trial by Qiao et al. Thirty patients with osteoarthritis of the knee and medial femoral-tibia condylar abnormalities were randomized to one of three treatment regimens. Microfracture combined with hyaluronan and autologous human adipose-derived mesenchymal progenitor cells injection resulted in long-term clinical improvement [47]. Kim et al. conducted a comparable clinical trial; except in this case, regenerative medicine was employed with slightly elevated tibia osteosynthesis. Clinical improvements and cartilage regeneration evaluations showed that the combination of autologous adipose tissue-derived mesenchymal stem cells and allogeneic cartilage from fresh cadavers produced better results [48].

Other clinical trials looked into tissue engineering techniques that did not require surgical intervention. In 2020, Garza and colleagues performed a trial on 39 patients with symptomatic knee osteoarthritis injected autologous stromal vasculature portion achieved by minor rhinoplasty harvest into the joint. Two dosages of stromal vascular fraction were compared to placebo injections in this clinical trial, and the results demonstrated a 12-month follow-up found a dose-based reduction in pain, but no change in cartilaginous-like structure [49].

The safety and efficacy of intra-articular injections of allogeneic human adipose-derived mesenchymal progenitor cells were investigated in a comparable clinical trial. Lu et al. studied 19 symptomatic intraregional knee osteoarthritis patients who received two injections of three different dosages of allogeneic human adipose-derived mesenchymal progenitor cells and found that despite improvement in health status, however, only those in the lowest quartile demonstrated slight articular cartilage regeneration [50]. Yoon et al. looked at seven patients with ICRS Grade 3 or 4 articular lesions in their knees using chondrocyte-based 3D microsphere type as a cell source. A 60-month follow-up revealed improved clinical outcomes and hyaline cartilage recovery. The absence of agreement on the optimal cell-based cartilage tissue engineering technique is due to information overload, conflicting outcomes, and the lack of follow-up studies. Furthermore, the effectiveness of stem cells is debatable, as their advantage over chondrocytes has yet to be established, and their use in the absence of physical and chemical stimuli has failed to produce satisfactory results [51]. All of the previously mentioned uncertainties are not assisting researchers in focusing their efforts in the appropriate direction. Scaffold-free techniques have shown promising results [51]. We think that scaffolds will still play an important role in supporting cells and giving the right signals for hyaline cartilage regrowth no matter what [51].

Cole et al. used an industrial cartilage allogeneic-graft as platform which are assistant cells released after microfracture in a recent prospective cohort research. This study comprised 48 patients with symptomatic localized cartilage abnormalities in the knees, and the results showed favorable clinical outcomes after enhanced microfracture at a 2-year follow-up [52]. Wolf et al. used a comparable approach in a clinical trial, using microfracture in combination with photoreactive chondroitin-sulfate/hyaluronan hydrogel. They observed 18 patients with full-thickness femoral condyle defects in the knee for 24 months following surgery in their clinical trial, and the hydrogel predictably demonstrated biocompatibility and efficacy by increasing the structural remodeling of articular cartilage defects [53]. Lee et al. again used microfracture augmentation, but this time, they used an adjunct made up of atelocollagen, thrombin, and fibrinogen to treat osteochondral lesions. The study included 60 patients with osteochondral lesion of the talus, and while both groups, control and experimental, showed clinical improvement, the quality of regenerated cartilage was greater with microfracture with atelocollagen augmentation [54]. Kim et al. conducted another clinical trial, comparing microfracture to the porcine-derived collagen-augmented chondrogenesis method in 100 patients with cartilage abnormalities in the knee, including those with osteoarthritis. After a 2-year follow-up, the porcine-derived C-ACT resulted in a better filling of the articular cartilage defects [55]. Only a few previous studies, on the other hand, looked at cell-free tissue engineering grafting approaches on their own. Efe et al. performed a similar experimental study in which cell-free collagen type I-based scaffolds were press-fit into 15 patients, and the results showed satisfactory clinical and imaging outcomes at 2-year follow-up [56]. Furthermore, Gupta et al. are currently conducting a prospective cohort research to assess using umbilical cord-derived Wharton's jelly as a source of proregenerative biochemical components to treat Kellgren and Lawrence grade 2 and 3 knee osteoarthritis [57].

For patients with early osteoarthritis who are not finding relief from nonsurgical treatment, hand surgeons should consider using autologous fat grafting as a viable alternative. In their study [58], Herold et al. confirmed the positive results in 50 patients with thumb carpometacarpal joint osteoarthritis (Eaton and Littler stage II–IV). After conservative measures failed, the intra-articular injection of processed fat (Coleman technique) improved grip and pinch strength at 12 months. They found that patients in stage II had significantly better outcomes than those in stages III and IV [59]. The largest cohort of patients available to date was published by Haas et al., who performed fat grafting in 99 first carpometacarpal joints and published their findings. In their study, they discovered that pain under stress was significantly lower at 2 and 6 weeks, as well as 3, 6, and 12 months, compared to baseline. Furthermore, scores on the Michigan Hand Outcome Questionnaire were significantly higher at six weeks, three months, six months, and twelve months. The strength of the pinch and grip was found to be unchanged after 12 months [60]. Kemper and colleagues found that mean values had decreased at 24 months when compared to the preoperative assessment. Interestingly, while pain and function improved during the first few months after surgery, complete satisfaction did not occur until 7 to 12 months after the procedure. We measured the strength of both hands, with a percentage difference between the treated and nontreated hand being displayed on the graph [60].

Adipose-derived stem cells can be seeded on biocompatible scaffolds or biomaterials to produce bone regeneration grafts using bone tissue engineering methods [61]. An implanted bioactive glass with adipose-derived stem cells was found to stimulate radiologically and histologically evident bone regeneration in animal models with critical size calvaria defects, as demonstrated by Saçak et al. [62]. In *in vitro* and in maxillofacial patients with malar augmentation procedures, seeded adipose-derived stem cells appear to be an excellent biomaterial capable of driving bone regrowth and remodeling in the hydroxyapatite-collagen hybrid scaffold [63].

The outcomes of recent cartilage tissue engineering clinical studies are presented in Table 2.

## 6. Conclusion

Articular cartilage tissue design, a comparatively recent scientific field, has made significant progress in the last two decades, but there are still many obstacles to practical application. Translatability and reproducibility are absent from the design of articular cartilage tissue in the seat-to-bedside process, and this is reflected in the inability of translational examination to overcome this barrier. Endoplasmic reticulum or mitochondrial breakdown, apoptosis, or excessive production of reactive oxygen species are all possible outcomes of chondrocyte passing. An additional problem is that changes in articular cartilage caused by physiological maturation and changes in the extracellular matrix are often not clearly distinguished. Extracellular matrix-related cartilage irregularities may necessitate the use of multiplex tissue design techniques. When it comes to joints other than the knee, arthroscopy perceptions and articular cartilage desert outer extension are the only tools available. Since models with actual deformities are used regardless of the climate in which they are studied, *in vitro*, *in vivo*, and clinical studies on articular cartilage sores prior to osteoarthritis progression seem pointless. In addition, a number of large-scale clinical preliminary and long-term follow-up studies, including the requirement for replication of *in vitro*, *ex vivo*, and clinical *in vivo* examinations, are needed. Long-term recovery is more important than short-term improvements in clinical symptoms and radiologic evaluations. In addition to the previously mentioned challenges, focusing on accessibility now presents new ones. Cell-based tissue design is extremely difficult because of the enormous number of studies and a wide range of tissue design parts and advancements. A lack of studies, a focus on collagen type I platforms, and information on the hidden tools of activity and advantages it has over cell-based tissue design and other existing intercessions are all problems with non-cell-based tissue design. Adaptability and clinical interpretation of these techniques are directly affected by other factors that influence their adaptability and microenvironment. Recreating the articular cartilage microenvironment is certain to show powerful cartilage recovery. Although there are some disadvantages, the design of articular cartilage tissue holds a lot of promise for the repair and prevention of cartilage degradation.

## Figures and Tables

**Table 1:** Representative of advantages and disadvantages of articular cartilage tissue engineering approaches.

Articular cartilage tissue engineering approaches	Advantages	Disadvantages
Scaffold-dependent approaches	(i) Provide 3D-microenvironment which is mimicking native articular cartilage tissue structure	(i) The long-term safety of the scaffold
	(ii) Promote cell growth and differentiation and deliver bioactive molecules that promote chondrogenesis	(i) Undefined degradation rate
	(iii) Mimic the articular cartilage's mechanical properties	(iii) Potential toxic degradation of byproducts
		(iv) Potential of immune resistance

Injectable-dependent approaches	(i) Cells can be delivered to the defect site only	(i) Undefined degradation rate
	(ii) Minimally invasive or noninvasive surgical procedures for articular cartilage regeneration	(ii) Potential toxic degradation of byproducts
		(iii) Potential of immune resistance
		(iv) No immediate structural and biomechanical alteration

Cell sheet approaches	(i) Extensive cellular resources and a rapid proliferative rate and capacity for chondrogenic differentiation	(i) No immediate structural and lack of the articular cartilage's mechanical properties
	(ii) No immune resistance	(ii) Potential disease transmission
	(iii) Promotes proliferation and accelerates chondrogenesis	(iii) Limitations in clinical trials experiments

**Table 2:** Reprehensive clinical studies' outcomes.

Clinical studies	Outcomes	Reference
Infrapatellar fat pad	Both *in vitro* and *in vivo* studies using Sprague Dawley rat osteochondral defect models in cartilage regeneration demonstrated better recovery	30
Intra-articular injection of autologous stromal vascular fraction	It is possible that E7-Exo delivered KGN-enabled *in situ* chondrogenesis could lead to an advanced stem cell therapy for osteoarthritis	32
Autologous Chondrocyte implantation (ACI)	According to the study results, long-term clinical improvement (more than 12 months postsurgery) can be achieved by combining microfracture with HA and autologous cells injection	47
Collagen type I- based scaffolds	Improved articular cartilage defect structural remodeling was achieved by using a biocompatible hydrogel	52–55

## Data Availability

The data that support the findings of this study are available on request from the corresponding author.
